# Hospital Festivals and Meetings

**Published:** 1895-04-20

**Authors:** 


### HOSPITAL FESTIVALS AND MEETINGS.

### HOSPITAL FOR DISEASES OF THE THROAT.

The annual general meeting was held at 20, Hanover Square, in the rooms of the Royal Medical and Chirurgical Society, on Wednesday, April 10th, the Duke of Sutherland in the chair. The Committee of Management reported an increase in the number of in-patients treated during the year. Subscriptions and donations had also slightly increased, and a legacy of £1,000 from Miss Grace Rolleston was thankfully acknowledged. The money now available for the extension of the hospital was thus raised to a sum of £3,500, and the committee hoped a further sum of £1,500 might soon be raised, when the building could at once be put in hand. The Duke of Sutherland, in moving the adoption of the report, made an appeal for the additional funds required, and Mr. Courtney Welsh, the chairman of the Hospital Committee, urged the same cause, explaining that the accommodation being now the same as when patients were far fewer in number, great inconvenience resulted, and the only course to be pursued was to add to the buildings. Land had been purchased, and only the already-mentioned £1,500 were needed in order to begin the work. The usefulness of the hospital would be immensely extended could these necessary improvements be effected forthwith. The meeting closed with the usual votes of thanks.

